# Survival of *Salmonella* in Tea Under Different Storage Conditions and Brewing Methods

**DOI:** 10.3389/fmicb.2022.816667

**Published:** 2022-03-18

**Authors:** Aiying Shi, Shenmiao Li, Hui Ma, Xin-Jun Du, Shuo Wang, Xiaonan Lu

**Affiliations:** ^1^Key Laboratory of Food Nutrition and Safety, Ministry of Education of China, Tianjin University of Science and Technology, Tianjin, China; ^2^Department of Food Science and Agricultural Chemistry, Faculty of Agricultural and Environmental Sciences, McGill University, Sainte-Anne-de-Bellevue, QC, Canada; ^3^Tianjin Key Laboratory of Food Science and Health, School of Medicine, Nankai University, Tianjin, China

**Keywords:** *Salmonella*, food-safety, viable but non-culturable, quantitative PCR, propidium monoazide, low-moisture

## Abstract

*Salmonella* is one of the leading causes of bacterial gastroenteritis. High prevalence of *Salmonella* in environment is partially due to its ability to enter the “viable but non-culturable” (VBNC) state when they encounter unfavorable conditions. Dried teas are traditionally believed to have a low risk of causing salmonellosis. This study investigated the survival of *Salmonella* in four types of dried teas under different storage conditions and brewing methods. A method that coupled propidium monoazide (PMA) and quantitative PCR was optimized to quantify VBNC *Salmonella* cells to assess the risk of *Salmonella* contamination in teas after brewing. Each tea sample was inoculated with *Salmonella* at an 8 log CFU/ml concentration and stored at 4, 10, and 25°C. Under three storage conditions, the number of survived *Salmonella* was highest in teas stored at 4°C and lowest in teas stored at 25°C. After storage of 120 days, culturable *Salmonella* was detected from all samples ranging from 6–7 log CFU/g (4°C storage) to 3–4 log CFU/g (25°C storage). The effectiveness of brewing methods in inactivating *Salmonella* was assessed by brewing inoculated teas at room temperature, 55, 75, and 100°C for 10 min. Brewing teas at 75 and 100°C significantly (*P* < 0.05) reduced the number of viable *Salmonella*, but VBNC *Salmonella* formed when brewed at 75°C. Altogether, *Salmonella* can persist in dried teas for over 3 months at a temperature ranging from 4 to 25°C, and thermal treatment delivered during home brewing may not eradicate *Salmonella* in teas.

## Introduction

Each year, consumption of unsafe food is responsible for over 600 million foodborne illnesses and 420,000 deaths worldwide (World Health Organization, 2015). Of the 31 identified etiologic agents of foodborne diseases, non-typhoidal *Salmonella enterica* serotypes are recognized as the bacterial agent with the highest capability to cause foodborne mortality (ca. 59,000 deaths). From a social-economic perspective, non-typhoidal *S. enterica* was considered the costliest foodborne pathogen among the fourteen major foodborne pathogens (Hoffmann et al., 2012; World Health Organization, 2015). *Salmonella* is highly prevalent in both agricultural and wildlife environments and can withstand a wide variety of stresses (Humphrey, 2004). Although most salmonellosis can be attributed to contaminated animal products (e.g., pork, poultry and eggs), a considerable number of worldwide *Salmonella*-associated outbreaks were linked to food with low moisture content, including spices, chocolate, peanut butter, almonds and paprika (Lehmacher et al., 1995; Kirk et al., 2004; Isaacs et al., 2005; Werber et al., 2005; Centers for Disease Control Prevention, 2007). In 2003, a nationwide *Salmonella* outbreak was associated with herbal tea in Germany, and the majority of the reported infections occurred in infants (Koch et al., 2005). In 2008, another *Salmonella* outbreak caused by herbal tea was reported in Serbia (Ilić et al., 2010). *Salmonella* is known to persist in desiccation conditions and respond effectively to environmental stimuli (Abdelhamid and Yousef, 2020). Several studies showed that desiccated *Salmonella* cells demonstrated a higher thermal resistance than untreated cells (Hiramatsu et al., 2005; Shachar and Yaron, 2006; Gruzdev et al., 2011). *Salmonella* can prevent water loss under suboptimal water activity conditions by osmoadaptation mechanisms, such as the production of intracellular osmoprotectants (Csonka and Hanson, 1991; Finn et al., 2013). Entering into a viable but non-culturable (VBNC) state has been proposed as another vital survival strategy of *Salmonella* under desiccation conditions (Oliver, 2010; Gruzdev et al., 2012). VBNC bacterial cells cannot be detected by the conventional culture-based methods, so that the risk of *Salmonella* contamination in low-moisture foods may be underestimated (Salive et al., 2020). Real-time quantitative PCR (qPCR) is an accurate and sensitive method to detect *Salmonella* in food matrices, but it cannot differentiate between live and dead bacteria and may overestimate the number of viable cells (Fittipaldi et al., 2011). An intercalating dye, propidium monoazide (PMA), has been identified to covalently bond to double-strand DNA upon light exposure and block the binding of DNA polymerase (Nocker et al., 2006). The bulky and positively charged PMA is membrane-impermeant that cannot enter viable cells with intact membranes (Nocker et al., 2006). Inclusion of PMA treatment prior to qPCR assay can selectively limit DNA amplification to the viable cells. The unbounded PMA are inactive during the light exposure and eliminated in the subsequent DNA extraction step.

Dried herbs and teas are widely traded and consumed globally. In recent years, *Salmonella* has implicated numerous recalls of herbs and teas. In the period of 2008–2011, European Commission triggered 22 alerts for herbs and species, and *Salmonella* was detected in 21 of these products (Public Health Ontario, 2015). In 2019, the Canadian Food Inspection Agency issued recalls for six dried tea products of different brands due to the potential risk of *Salmonella* contamination (Canadian Food Inspection Agency, 2019). Although the thermal treatment recommended by brewing instructions should inactivate *Salmonella*, consumers may use different brewing methods depending on their preferences. It is therefore important to assess the survival of *Salmonella* in the real-world scenarios of storage and brewing conditions for tea products. However, there are few studies on the survival of *Salmonella* in herbal tea and other tea products. Keller et al. (2015) investigated the survival of *Salmonella* in tea during storage and brewing using the conventional plating assay, but they did not assess the potential risks of VBNC *Salmonella* that may form due to the stresses induced upon brewing. This study aims to investigate the survival of *Salmonella* in four types of tea products stored and brewed at different temperatures that mimic in-home operations.

## Materials and Methods

### Bacterial Strains and Culture Conditions

Four *Salmonella* strains, namely *S. enterica* serovar Enteritidis (CMCC 50041), *S.* Typhimurium (CMCC 50115), *S.* Agona (ATCC^®^51957*™*) and *S*. Newport (ATCC^®^6962*™*), were used for investigating the survival of *Salmonella* in tea products. Dehydrated culture media, TSA and TSB, were purchased from Beijing Solarbio Science and Technology Co., (Beijing, China). The specificity of qPCR was verified using thirteen bacterial strains, including Gram-negative and Gram-positive bacteria (Table 1). All bacterial strains were routinely cultivated on Tryptic Soy agar (TSA) plates at 37°C under aerobic conditions for 16 h. Liquid cultures used as bacterial inoculants were obtained by growing a single colony of each strain in Tryptic Soy broth (TSB) with constant shaking at 37°C under aerobic conditions for 4 h. The bacterial cells were harvested by centrifugation at 8,000 × *g* for 5 min, and cell pellets were washed three times by phosphate-buffered saline (PBS). The washed bacterial cells were resuspended in PBS to reach a final concentration of 10^8^ CFU/ml. Equal volumes of bacterial suspension of each strain were mixed to make inoculant cocktails at a 10^8^ CFU/ml final concentration. The prepared inoculant was stored at 4°C for 24 h prior to inoculation.

**TABLE 1:** Bacterial strains used for qPCR specificity tests.

Species	Strain	qPCR results
*Salmonella enterica* serovar Enteritidis	CMCC 50041	+
	ATCC 51957	+
	ATCC 6962	+
*Salmonella enterica* serovar Typhimurium	CMCC 50115	+
*Cronobacter sakazakii*	ATCCBAA-894	−
	ATCC29544	−
*Cronobacter muytjensii*	ATCC 51329	−
*Cronobacter malonaticus*	SAKA 10310	−
*Escherichia coli*	ATCC 10305	−
*Enterobacter cloacae*	ATCC 23373	−
*Staphylococcus aureus*	ATCC25923	−
*Staphylococcus saprophyticus*	ATCC 49907	−

### Collection and Preparation of Tea Samples

Four types of loose-leaf teas, namely black tea, green tea, peppermint tea, and chamomile tea, were purchased from local supermarkets. All the tea samples were originally vacuum sealed by the manufacturers. The microbial background of all tea products was determined by the plating assay prior to further experiments to exclude highly contaminated samples. Briefly, 3 g of tea were added to 27 ml sterile PBS and mixed thoroughly, and 0.1 ml of the supernatant was spread onto TSA plates, followed by incubation at 37°C for 24 h. The products with no culturable bacteria were selected and further sterilized at 121°C for 15 min. The absence of culturable cells was verified by the plating assay.

### Preparation of Live and Dead Bacterial Cultures

Live bacterial cultures were prepared by mixing 4 *Salmonella* overnight cultures to achieve a final concentration of 10^8^ CFU/ml in PBS as aforementioned in section “Bacterial Strains and Culture Conditions.” Dead bacterial cultures were collected by heating the live bacterial cultures at 100°C for 10 min in boiling water. The viability of the dead bacterial culture was verified by the plating assay.

### PMA Optimization and DNA Extraction

Propidium monoazide concentration, dark incubation time, and light exposure time were optimized by orthogonal experiments. One millimolar PMA stock solution was prepared by dissolving PMA (Biotium Inc., Hayward, CA, United States) in 20% dimethyl sulfoxide (Sigma-Aldrich) according to the manufacturer’s instruction and then stored at −20°C in dark until use. The prepared live and dead bacterial cultures at 10^8^ CFU/ml were treated with PMA at a range of different concentrations. Dark incubation of PMA and bacteria mixtures was performed at room temperature with constant shaking at 100 rpm, followed by photoactivation under a 650-W halogen light at a distance of 20 cm from the samples. PMA treated cells were then washed three times with sterile distilled water, and the genome DNA (gDNA) was extracted using TIANamp Bacterial DNA Extraction Kit (Tiangen, Beijing, China). The optimal condition of PMA treatment was defined as the condition that can entirely prevent qPCR signals from dead bacterial cells (Ct value > 35, higher is better) without affecting the viability of live bacterial cells (Ct < 35, lower is better).

To determine the optimal concentration of PMA for discriminating between live and dead bacterial cells, six different PMA concentrations, namely 0, 5, 10, 20, 50 and 100 μM, were used to treat prepared live and dead *Salmonella* cultures separately. Dark incubated time was 10 min, and photoactivation time was 10 min. To determine the optimal time of dark incubation, PMA at a concentration of 20 μM was used to treat live and dead *Salmonella* cultures. Five dark incubation times were tested, namely 0, 5, 10, 15 and 20 min. The photoactivation time was 10 min. PMA at a concentration of 20 μM was used to treat live and dead *Salmonella* cultures to determine the optimal time of photoactivation. The dark incubation time was 5 min. Five light exposure times were tested, namely 0, 5, 10, 15, and 20 min.

### PMA-qPCR Assay

The primers targeting the *ttrA* gene of *Salmonella* specific *ttr* locus were used for *Salmonella* quantification as previously described (Martin et al., 2012). The sequences of the primers were *ttrA* forward (5′-GTCGCAGGAACACCCGATT-3′) and *ttrA* reverse (5′-TTGCTGCCGAAGCTATTTAGC-3′), and the amplicon was 417 bp. The qPCR amplification was performed in a 20-μl reaction system, containing 2.0 μl of template DNA, 10 μl of TB Green Premix Ex Taq II (Tli RNaseH Plus, Takara, Dalian, China), 200 nM of each primer, 0.4 μl of ROX Reference Dye, and 6.0 μl of sterile distilled water. The qPCR analysis was conducted using a Mastercycler ep gradient realplex system (Eppendorf, Germany) *via* the following program: an initial denaturation at 95°C for 2 min, 40 cycles of denaturation at 95°C for 15 s, annealing at 64.5°C for 30 s and elongation at 68°C for 60 s. A non-template negative control was included in each run of qPCR. The experiment was conducted at least in triplicates. The specificity of PMA-qPCR was tested using 13 different bacterial strains (Table 1).

### Standard Curves and Amplification Efficiency

The sensitivity and amplification efficiency of the optimized PMA-qPCR assay in testing *Salmonella* in each type of tea product was separately determined. *Salmonella* cultures at the exponential phase were used to develop a series of 10-fold dilutions ranging from 1 to 8 log CFU/ml. The standard curves were established by plotting the concentration of *Salmonella* cells against the obtained Ct values. The slope of each standard curve was used to calculate the amplification efficiency (*E*) of PMA-qPCR for the specific type of tea using the equation *E* = [10^(–1/slope)^ − 1] × 100%.

### Inoculation of Tea Products

Four types of tea products were weighed and transferred into sterile Petri dishes aseptically in quantities of 3 g. One milliliter of prepared *Salmonella* inoculant (section “Bacterial Strains and Culture Conditions”) was evenly pipetted onto the aliquoted tea samples. The inoculated tea samples were dried in biological safety cabinet for 24 h prior to further analysis. The re-dried tea samples were then vacuum sealed in small sterile sample bags (12 cm × 18 cm) using a vacuum sealer (Yute, Shanghai, China). To study the influence of temperature on the survival of *Salmonella*, the sealed samples were stored at three different temperatures, namely 4, 10, and 25°C.

### Survival of *Salmonella* During Storage

The number of culturable *Salmonella* in 4 tea samples was separately determined after sealing (day 0) and after storage for 5, 10, 20, 30, 60, 90, and 120 days at 4, 10, and 25°C. At each sampling point, population of culturable *Salmonella* was determined by the plating assay. Briefly, 3 g of tea were added to 27 ml of sterile PBS and mixed thoroughly, and 1 ml of the supernatant was collected and serially diluted. The number of microbes was enumerated by spreading 0.1 ml of each dilution onto TSA plates, followed by incubation at 37°C for 24 h.

### Survival of *Salmonella* After Brewing

Survival of *Salmonella* after brewing at three temperatures, namely 55, 75, and 100°C, was determined. Four types of tea samples were inoculated and dried as aforementioned in section “Inoculation of Tea Products.” Three grams of each inoculated tea sample was aseptically transferred into Erlenmeyer flasks (250 ml), and 100 ml of preheated water at 55, 75, and 100°C was added to the flasks, separately. The flasks were held at room temperature for 10 min in a biological safety cabinet. The number of culturable *Salmonella* was determined by the plating assay. One milliliter of the tea supernatant was collected for DNA extraction and PMA-qPCR analysis as described in section “PMA-qPCR Assay” to determine the number of VBNC bacterial cells.

### Statistical Analysis

All experiments were conducted at least in three replicates. Results were presented as the mean ± standard deviation. Data analysis and visualization were performed using Origin (version OriginPro 2020, OriginLab Corporation, United States). One-way ANOVA followed by Duncan’s test was used to determine if the difference was statistically significant (*P* < 0.05).

## Results and Discussion

### Optimization of Propidium Monoazide Treatment Condition

Propidium monoazide treatment has been reported to effectively prevent the indiscriminate amplification of bacterial DNA from viable and dead cells (Nocker et al., 2006; Josefsen et al., 2010). Previous studies demonstrated the capability of PMA in restricting DNA amplification to viable *Salmonella* cells in food matrices (Liang et al., 2011; Han et al., 2020). However, the optimal condition of PMA treatment varied widely among different studies. It is necessary to optimize the factors that can affect the efficiency of PMA treatment, including PMA concentration, dark incubation time, and light exposure time (Udomsil et al., 2016; Yuan et al., 2018). Insufficient concentration of PMA may not be able to suppress DNA amplification from dead cells entirely, and the number of viable cells can be overestimated.

**FIGURE 1:**
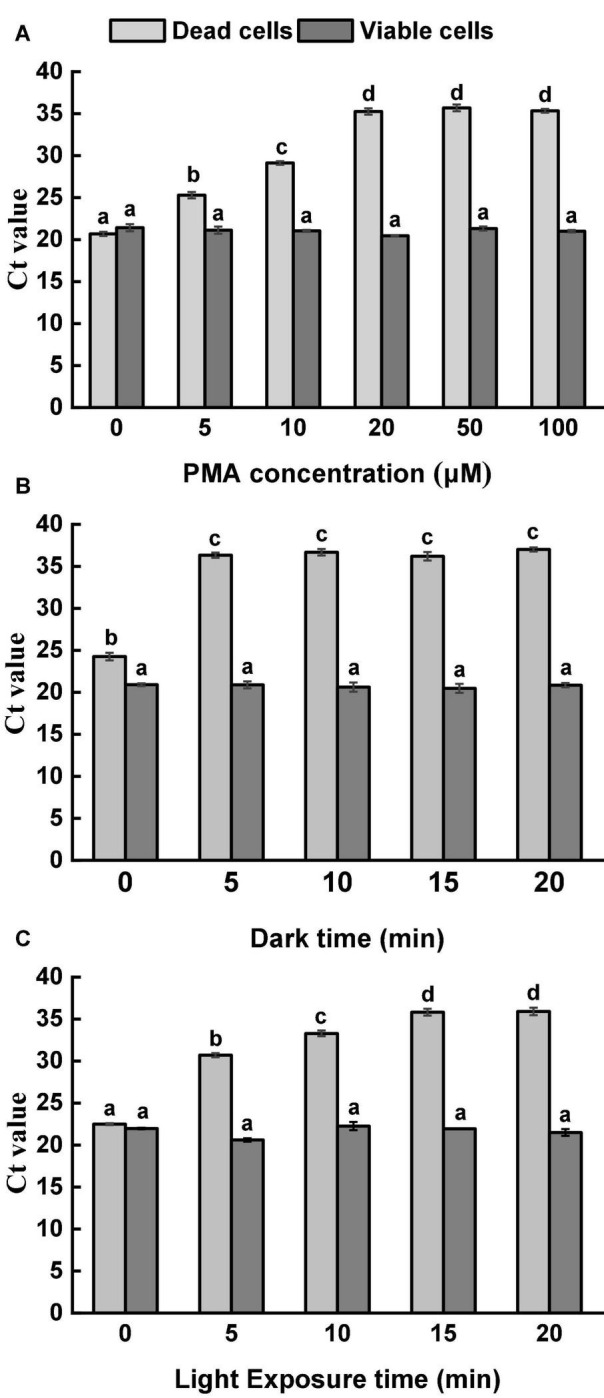
Optimization of PMA treatment condition. **(A)** Optimization of PMA concentration. Viable and dead *Salmonella* cells were separately treated with PMA at a range of concentrations, followed by 10 min dark incubation and 10 min light exposure. **(B)** Optimization of dark incubation time. Viable and dead *Salmonella* cells were separately treated with PMA at a concentration of 20 μM. Samples were incubated in dark for different durations, followed by light exposure for 10 min. **(C)** Optimization of light exposure time. Viable and dead *Salmonella* cells were separately treated with PMA at a concentration of 20 μM. Samples were incubated in dark for 5 min, followed by light exposure at different durations. Different letters indicated significant differences among different groups (*P* < 0.05).

Moreover, PMA at a high concentration showed cytotoxicity to viable cells. In the current study, five different PMA concentrations were applied to treat viable and dead *Salmonella* cells at the concentration of 10^8^ CFU/ml. Figure 1A demonstrates the results of the optimization of PMA concentration. For viable cells, similar Ct values were obtained from all tested PMA concentrations, indicating that the viability of *Salmonella* was not influenced even at the highest tested concentration. For thermal-inactivated cells, Ct values increased along with the increase of PMA concentrations up to 20 μM. There was no significant difference in the obtained Ct values among the treatments of 20, 50, and 100 μM PMA. Therefore, 20 μM PMA was determined as the optimal concentration to inhibit DNA amplification in dead cells effectively.

With the optimized PMA concentration, dark incubation and light exposure durations were further adjusted (Figures 1B,C). DNA amplification was neither significantly affected by dark incubation time nor light exposure time for viable *Salmonella* cells. For dead *Salmonella* cells, dark incubation for 5 min and light exposure for 15 min were considered sufficient for PMA penetration and binding. Based on the results, 20 μM PMA, dark incubation for 5 min, and light exposure for 15 min were selected as the optimal PMA treatment condition and used for further analysis.

### PMA-qPCR Assay

**FIGURE 2:**
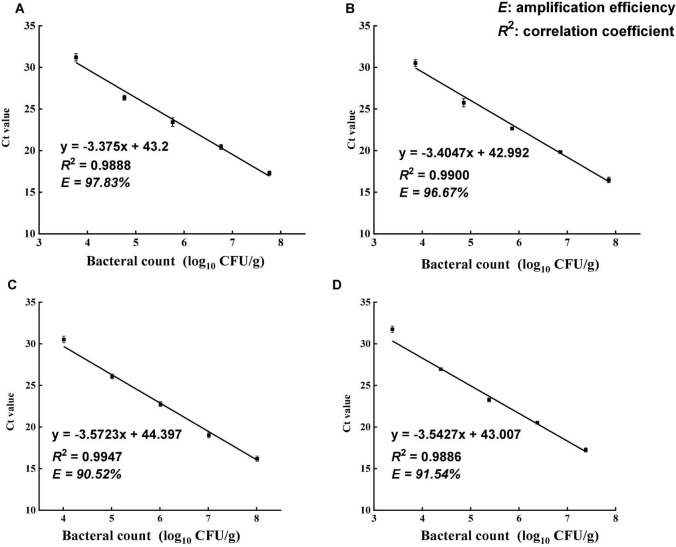
Standard curves of PMA-qPCR for the quantification of viable *Salmonella enterica* in four types of teas. **(A)** Black tea. **(B)** Green tea. **(C)** Peppermint tea. **(D)** Chamomile tea. The standard curves were generated from 10-fold dilutions of viable *Salmonella* cells in the supernatant of each tea. Amplification efficiency (*E*) of PMA-qPCR assay for each tea matrix was calculated using equation *E* = [10^(–1/slope)^ – 1] × 100%.

The specificity of the primer set was tested using 13 different bacterial strains (Table 1). All *Salmonella* strains showed positive results in DNA amplification, while no amplification was observed for non-*Salmonella* species. The *ttrA* primer set was highly specific to *Salmonella*, consistent with a previous study (Martin et al., 2012). Standard curve is required to estimate the amplification efficiency of qPCR reaction and quantify the number of cells in the samples. Desired amplification efficiency should be between 90 and 110% (Svec et al., 2015). Amplification efficiency can be affected by several factors, including variations in instrument, integrity of templates, inaccurate dilution, and presence of PCR inhibitors (Svec et al., 2015). Several studies demonstrated that high turbidity and presence of dark particles (e.g., clay, silt, and sludge) in samples can decrease PMA-qPCR efficiency (Bae and Wuertz, 2009; Li et al., 2014; Desneux et al., 2015). Tea is a complex matrix that contains many phytochemicals, pigments, and possibly dust so that the inhibitory effects of tea samples were addressed by conducting matrix-matched standard curves that is specific to each type of tea. The correlation coefficients for all four types of tea matrices were higher than 0.98, and the amplification efficiency ranged between 90 and 110% (Figure 2). The applicability of PMA-qPCR to detect and quantify *Salmonella* in four types of tea samples was thus confirmed.

### Survival of *Salmonella* During Storage

**FIGURE 3:**
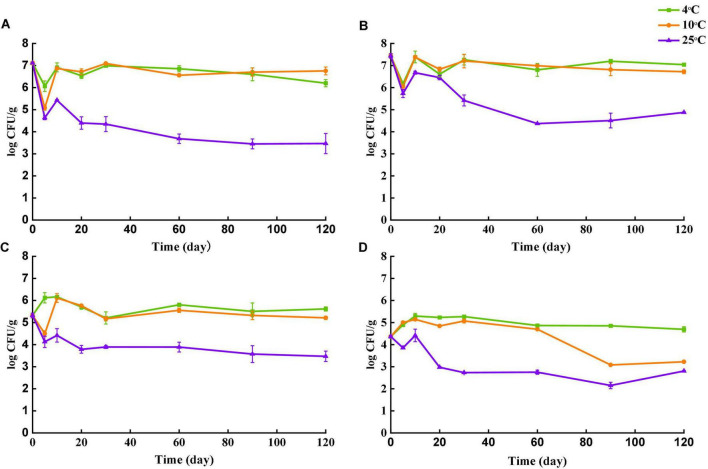
Survival of *Salmonella* in four types of teas during storage at 4, 10, and 25°C. **(A)** Black tea. **(B)** Green tea. **(C)** Peppermint tea. **(D)** Chamomile tea. Number of culturable *S. enterica* was monitored at day 0, 5, 10, 20, 30, 60, 90 and 120.

Survival of *Salmonella* in four types of tea over the storage of 120 days at different temperatures is shown in Figure 3. The initial population of *Salmonella* in each type of tea was assessed after inoculation by the plating assay. Despite the same inoculant used, amount of *Salmonella* cells recovered were different among different tea types. Initial population recovered from black tea and green tea was 7.11 log CFU/g and 7.44 log CFU/g, respectively. The number of *Salmonella* for peppermint and chamomile on day 0 was 5.31 log CFU/g and 4.37 log CFU/g, respectively. The differences in the recovery rate could be caused by different natures of tea matrices. Food with large particles and porous structures may entrap bacteria (Stevens and Jaykus, 2004). The lowest *Salmonella* recovery was observed for chamomile tea, which is a flower rather than leave. The flowers may have more niches to trap *Salmonella* cells upon their settlement, resulting in insufficient separation during sample preparation.

The number of survived *Salmonella* in all teas was stable when stored at 4 and 10°C. The largest decline of culturable *Salmonella* was observed in samples stored at 25°C for all types of tea. Despite the overall decrease trend of culturable *Salmonella* in all four types of tea over the 120 days, fluctuations in *Salmonella* cell numbers were observed at the early stages. The population of *Salmonella* in black tea and green tea declined rapidly during the first 5 days regardless of temperature, but the number of culturable *Salmonella* recovered almost to the initial level on day 10 except the samples stored at 25°C. For storage at 4°C, the number of culturable *Salmonella* increased in peppermint and chamomile tea untiled day 10, followed by a gradual decrease. At 120 days after inoculation, samples stored at 25°C showed the lowest culturable *Salmonella* counts for all four types of tea, indicating that *Salmonella* survived better in teas at 4°C than 25°C. A similar trend was reported in peanut butter that the number of survived *Salmonella* was higher in samples stored at 4°C than that at 25°C (Burnett et al., 2000). Another study conducted by Keller et al. (2015) compared the survival of *Salmonella* in tea and demonstrated that the decline of viable *Salmonella* was faster in tea samples stored at 35°C than that at 25°C. Two studies conducted by Beuchat and Mann (2010, 2014) also demonstrated that survival of *Salmonella* in low-moisture food was favored at refrigeration temperature rather than at room temperature. *Salmonella* can respond to various environmental stressors by reducing metabolism and even entering a metabolically dormant state (Humphrey, 2004). Low temperature as a stimulus may trigger the stress responses of bacteria at an early stage and enhance the subsequent survivals during long-term storage.

### Survival of *Salmonella* After Brewing

**FIGURE 4:**
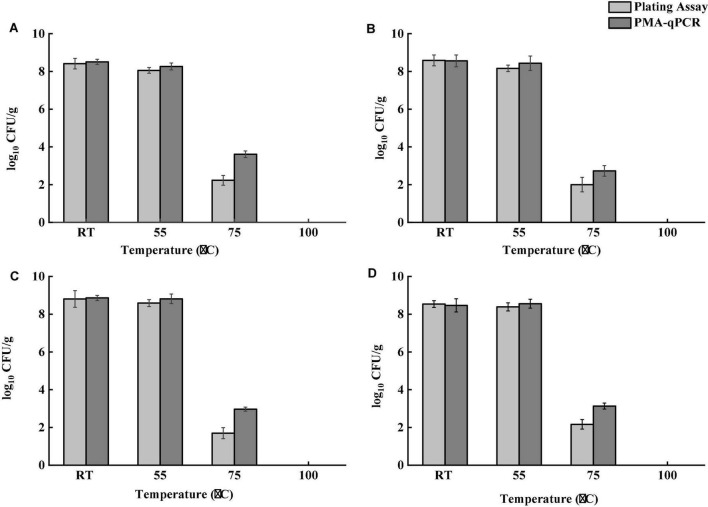
Survival of *Salmonella* in four types of teas after brewing at room temperature (RT), 55, 75, and 100°C for 10 min. **(A)** Black tea. **(B)** Green tea. **(C)** Peppermint tea. **(D)** Chamomile tea. Total number of viable *S. enterica* was determined by PMA-qPCR, and total number of culturable *S. enterica* was determined using the plating assay.

Tea products generally undergo a dehydration process during production. *Salmonella* contamination may occur before dehydration. *Salmonella* cells pre-exposed to a desiccation condition develop tolerance to multiple stresses, including heat (Gruzdev et al., 2011). Besides, both thermal and osmotic stresses induced during brewing may lead to the formation of VBNC bacterial cells. Although VBNC *Salmonella* cells are generally avirulent, they could resuscitate and regain virulence under favorable conditions (Highmore et al., 2018). An effective brewing process should be able to eliminate both culturable and VBNC *Salmonella*. To determine the effect of different brewing temperatures on *Salmonella*, four different temperatures were applied. Figure 4 demonstrates the survival of *Salmonella* after brewing as determined by both the conventional plating assay and PMA-qPCR. Tea brewed at room temperature and 55°C showed no significant difference in *Salmonella* cell counts for all teas samples. The difference of *Salmonella* cell counts determined by the plating assay and PMA-qPCR was not statistically significant (*P* > 0.05) for all teas samples brewed at room temperature and 55°C. Brewing teas achieved more than 8-log reduction of *Salmonella* at 100°C for 10 min. At 75°C, the number of viable *Salmonella* cells determined by PMA-qPCR was significantly higher (*P* < 0.05) than that obtained by the plating assay for all tea samples. Taken together, the plating assay might underestimate viable *Salmonella* in teas after brewing. Although brewing tea at 100°C was validated to be effective in inactivating *Salmonella* in teas, it is unlikely to be a common operation for home-brewing. Therefore, *Salmonella* in teas can pose a potential risk to consumers if insufficient thermal treatment is delivered.

## Conclusion

*Salmonella* can survive in teas for over 3 months at a wide range of temperatures. Storing teas at refrigeration temperature could enhance the survival of *Salmonella* instead of eliminating it, and brewing cannot inactivate *Salmonella* in teas at a temperature below 55°C. Thus, teas contaminated with *Salmonella* can pose a risk to consumers after long-time storage and brewing.

## Data Availability Statement

The original contributions presented in the study are included in the article/supplementary material, further inquiries can be directed to the corresponding author/s.

## Author Contributions

AS and SL performed the experiment and wrote the manuscript. HM contributed to the experiment. X-JD, SW, and XL designed the project. XL edited the writings of the manuscript. All authors contributed to the article and approved the submitted version.

## Conflict of Interest

The authors declare that the research was conducted in the absence of any commercial or financial relationships that could be construed as a potential conflict of interest.

## Publisher’s Note

All claims expressed in this article are solely those of the authors and do not necessarily represent those of their affiliated organizations, or those of the publisher, the editors and the reviewers. Any product that may be evaluated in this article, or claim that may be made by its manufacturer, is not guaranteed or endorsed by the publisher.

## References

[B1] Abdelhamid A. G. Yousef A. E. (2020). Collateral adaptive responses induced by desiccation stress in *Salmonella enterica*. *LWT* 133:110089.

[B2] Bae S. Wuertz S. (2009). Discrimination of viable and dead fecal Bacteroidales bacteria by quantitative PCR with propidium monoazide. *Appl. Environ. Microbiol.* 75 2940–2944. 10.1128/AEM.01333-08 19270114 PMC2681701

[B3] Beuchat L. R. Mann D. A. (2010). Factors affecting infiltration and survival of *Salmonella* on in-shell pecans and pecan nutmeats. *J. food Prot.* 73 1257–1268. 10.4315/0362-028x-73.7.1257 20615338

[B4] Beuchat L. R. Mann D. A. (2014). Survival of *Salmonella* on dried fruits and in aqueous dried fruit homogenates as affected by temperature. *J. food Prot.* 77 1102–1109. 10.4315/0362-028X.JFP-13-549 24988015

[B5] Burnett S. Gehm E. Weissinger W. Beuchat L. (2000). Survival of *Salmonella* in peanut butter and peanut butter spread. *J. Appl. Microbiol.* 89 472–477. 11021579 10.1046/j.1365-2672.2000.01138.x

[B6] Centers for Disease Control Prevention. (2007). Multistate outbreak of *Salmonella* serotype Tennessee infections associated with peanut butter–United States, 2006-2007. *MMWR Morb. Mortal. Wkly. Rep.* 56 521–524. 17538526

[B7] Csonka L. N. Hanson A. D. (1991). Prokaryotic osmoregulation: genetics and physiology. *Ann. Rev. Microbiol.* 45 569–606. 1741624 10.1146/annurev.mi.45.100191.003033

[B8] Desneux J. Chemaly M. Pourcher A.-M. (2015). Experimental design for the optimization of propidium monoazide treatment to quantify viable and non-viable bacteria in piggery effluents. *BMC Microbiol.* 15:164. 10.1186/s12866-015-0505-6 26276157 PMC4537567

[B9] Finn S. Condell O. McClure P. Amézquita A. Fanning S. (2013). Mechanisms of survival, responses and sources of *Salmonella* in low-moisture environments. *Front. Microbiol.* 4:331. 10.3389/fmicb.2013.00331 24294212 PMC3827549

[B10] Fittipaldi M. Codony F. Adrados B. Camper A. K. Morató J. (2011). Viable real-time PCR in environmental samples: can all data be interpreted directly? *Microb. Eco.* 61 7–12. 10.1007/s00248-010-9719-1 20632000

[B11] Gruzdev N. Pinto R. Sela S. (2011). Effect of desiccation on tolerance of *Salmonella enterica* to multiple stresses. *Appl. Environ. Microbiol.* 77 1667–1673. 10.1128/AEM.02156-10 21216905 PMC3067256

[B12] Gruzdev N. Pinto R. Sela S. (2012). Persistence of *Salmonella enterica* during dehydration and subsequent cold storage. *Food Microbiol.* 32 415–422. 10.1016/j.fm.2012.08.003 22986208

[B13] Han L. Wang K. Ma L. Delaquis P. Bach S. Feng J. (2020). Viable but nonculturable *Escherichia coli* O157: H7 and *Salmonella enterica* in fresh produce: rapid determination by loop-mediated isothermal amplification coupled with a propidium monoazide treatment. *Appl. Environ. Microbiol.* 86 e2566–e2519. 10.1128/AEM.02566-19 32005729 PMC7082562

[B14] Highmore C. J. Warner J. C. Rothwell S. D. Wilks S. A. Keevil C. W. (2018). Viable-but-nonculturable Listeria monocytogenes and *Salmonella enterica* serovar Thompson induced by chlorine stress remain infectious. *MBio* 9 e540–e518. 10.1128/mBio.00540-18 29666286 PMC5904417

[B15] Hiramatsu R. Matsumoto M. Sakae K. Miyazaki Y. (2005). Ability of Shiga toxin-producing *Escherichia coli* and *Salmonella* spp. to survive in a desiccation model system and in dry foods. *Appl. Environ. Microbiol.* 71 6657–6663. 10.1128/AEM.71.11.6657-6663.2005 16269694 PMC1287607

[B16] Hoffmann S. Batz M. B. Morris J. G. (2012). Annual cost of illness and quality-adjusted life year losses in the United States due to 14 foodborne pathogens. *J. food prot.* 75 1292–1302. 10.4315/0362-028X.JFP-11-417 22980013

[B17] Humphrey T. (2004). *Salmonella*, stress responses and food safety. *Nat. Rev. Microbiol.* 2 504–509. 10.1038/nrmicro907 15152206

[B18] Ilić S. Đurić P. Grego E. (2010). *Salmonella* Senftenberg infections and fennel seed tea. *Serbia. Emerg. Infect. Dis.* 16:893. 10.3201/eid1605.091555 20409401 PMC2954001

[B19] Canadian Food Inspection Agency (2019). Complete listing of all recalls and allergy alerts. *J Food Prot.* 82 1901–1908. 31633425 10.4315/0362-028X.JFP-19-235

[B20] Isaacs S. Aramini J. Ciebin B. Farrar J. Ahmed R. Middleton D. (2005). An international outbreak of salmonellosis associated with raw almonds contaminated with a rare phage type of *Salmonella* Enteritidis. *J. food prot.* 68 191–198. 10.4315/0362-028x-68.1.191 15690826

[B21] Josefsen M. H. Löfström C. Hansen T. B. Christensen L. S. Olsen J. E. Hoorfar J. (2010). Rapid quantification of viable Campylobacter bacteria on chicken carcasses, using real-time PCR and propidium monoazide treatment, as a tool for quantitative risk assessment. *Appl. Environ. Microbiol.* 76 5097–5104. 10.1128/AEM.00411-10 20562292 PMC2916463

[B22] Keller S. E. Stam C. N. Gradl D. R. Chen Z. Larkin E. L. Pickens S. R. (2015). Survival of *Salmonella* on chamomile, peppermint, and green tea during storage and subsequent survival or growth following tea brewing. *J. food prot.* 78 661–667. 10.4315/0362-028x.jfp-14-508 25836389

[B23] Kirk M. Little C. Lem M. Fyfe M. Genobile D. Tan A. (2004). An outbreak due to peanuts in their shell caused by *Salmonella enterica* serotypes Stanley and Newport–sharing molecular information to solve international outbreaks. *Epidemiol. Infect.* 132 571–577. 10.1017/s095026880400216x 15310157 PMC2870136

[B24] Koch J. Schrauder A. Alpers K. Werber D. Frank C. Prager R. (2005). *Salmonella* Agona outbreak from contaminated aniseed. *Germany. Emerg. Infect. Dis.* 11:1124. 10.3201/eid1107.041022 16022796 PMC3371796

[B25] Lehmacher A. Bockemühl J. Aleksic S. (1995). Nationwide outbreak of human salmonellosis in Germany due to contaminated paprika and paprika-powdered potato chips. *Epidemiol. Infect.* 115 501–511. 10.1017/s0950268800058660 8557082 PMC2271603

[B26] Li D. Tong T. Zeng S. Lin Y. Wu S. He M. (2014). Quantification of viable bacteria in wastewater treatment plants by using propidium monoazide combined with quantitative PCR (PMA-qPCR). *J. Environ. Sci.* 26 299–306. 10.1016/s1001-0742(13)60425-8 25076521

[B27] Liang N. Dong J. Luo L. Li Y. (2011). Detection of viable *Salmonella* in lettuce by propidium monoazide real-time PCR. *J. food sci.* 76 M234–M237. 10.1111/j.1750-3841.2011.02123.x 22417362

[B28] Martin B. Raurich S. Garriga M. Aymerich T. (2012). Effect of Amplicon Length in Propidium Monoazide Quantitative PCR for the Enumeration of Viable Cells of *Salmonella* in Cooked Ham. *Food Anal. Methods* 6 683–690. 10.1007/s12161-012-9460-0

[B29] Nocker A. Cheung C.-Y. Camper A. K. (2006). Comparison of propidium monoazide with ethidium monoazide for differentiation of live vs. dead bacteria by selective removal of DNA from dead cells. *J. Microbiol. Methods* 67 310–320. 10.1016/j.mimet.2006.04.015 16753236

[B30] Oliver J. D. (2010). Recent findings on the viable but nonculturable state in pathogenic bacteria. *FEMS Microbiol. Rev.* 34 415–425. 10.1111/j.1574-6976.2009.00200.x 20059548

[B31] Public Health Ontario (2015). *Case Study: Pathogens and Spices.* Harm, Us: Public Health Ontario

[B32] Salive A. F. V. Prudêncio C. V. Baglinière F. Oliveira L. L. Ferreira S. O. Vanetti M. C. D. (2020). Comparison of stress conditions to induce viable but non-cultivable state in *Salmonella*. *Braz. J. Microbiol.* 51 1269–1277. 10.1007/s42770-020-00261-w 32291740 PMC7455614

[B33] Shachar D. Yaron S. (2006). Heat tolerance of *Salmonella enterica* serovars Agona, Enteritidis, and Typhimurium in peanut butter. *J. food prot.* 69 2687–2691. 10.4315/0362-028x-69.11.2687 17133812

[B34] Stevens K. A. Jaykus L.-A. (2004). Bacterial separation and concentration from complex sample matrices: a review. *Crit. Rev. Microbiol.* 30 7–24. 10.1080/10408410490266410 15116760

[B35] Svec D. Tichopad A. Novosadova V. Pfaffl M. W. Kubista M. (2015). How good is a PCR efficiency estimate: Recommendations for precise and robust qPCR efficiency assessments. *Biomol. Detect. Quantif.* 3 9–16. 10.1016/j.bdq.2015.01.005 27077029 PMC4822216

[B36] Udomsil N. Chen S. Rodtong S. Yongsawatdigul J. (2016). Quantification of viable bacterial starter cultures of Virgibacillus sp. and Tetragenococcus halophilus in fish sauce fermentation by real-time quantitative PCR. *Food Microbiol.* 57 54–62. 10.1016/j.fm.2016.01.004 27052702

[B37] Werber D. Dreesman J. Feil F. Van Treeck U. Fell G. Ethelberg S. (2005). International outbreak of *Salmonella* Oranienburg due to German chocolate. *BMC Infect. Dis.* 5:7. 10.1186/1471-2334-5-7 15691371 PMC552305

[B38] World Health Organization (WHO) (2015). *WHO Estimates of the Global Burden of Foodborne Diseases: Foodborne Disease Burden Epidemiology Reference Group 2007-2015*: World Health Organization. Switzerland: World Health Organization

[B39] Yuan Y. Zheng G. Lin M. Mustapha A. (2018). Detection of viable *Escherichia coli* in environmental water using combined propidium monoazide staining and quantitative PCR. *Water Res.* 145 398–407. 10.1016/j.watres.2018.08.044 30173100

